# RE: Do no harm: can school mental health interventions cause iatrogenic harm?

**DOI:** 10.1192/bjb.2023.65

**Published:** 2023-10

**Authors:** Katherine Venturo-Conerly, Tom L. Osborn, Eve S. Puffer, John Weisz, Afra van der Markt

**Affiliations:** PhD student, Harvard University, Cambridge, Massachusetts, USA; and Co-Founder and Scientific Director, Shamiri Institute, Nairobi, Kenya; Founder and CEO, Shamiri Institute, Nairobi, Kenya; associate professor, Duke University Department of Psychology and Neuroscience, Duke Global Health Institute, Durham, USA; Professor of the Social Sciences, Department of Psychology, Harvard University, Cambridge, Massachusetts, USA; research consultant and psychiatrist, Shamiri Institute, Nairobi, Kenya; and Amsterdam UMC location VUmc, Psychiatry, Amsterdam, The Netherlands. Email: afra.vandermarkt@shamiri.institute

## Do good: minimising risk of harm in school-based interventions

This thoughtful and thought-provoking article emphasises potential iatrogenic harm for some students caused by school mental health interventions, particularly universal ones, which should be avoided.^1^ Although we agree, the advantages of school-based universal interventions for addressing the global shortage of specialised mental health caregivers should be considered. Most youths with mental illness do not receive treatment, especially in low- and middle-income countries, where only 5% of randomised controlled trials of youth psychotherapy have been conducted.^2^ Group-based interventions in convenient, low-stigma settings (e.g. schools) are a cost-effective way to reach the millions of adolescents with mental health concerns. We herein discuss how their harm may be minimised and propose directions for future research.

One likely mechanism of iatrogenic harm, mentioned by the authors, is students becoming more aware of existing symptoms without receiving sufficient information to gauge the severity of and address these symptoms. Isolated psychoeducational interventions increase awareness without providing necessary coping skills, posing a particular risk. Similarly, brief mindfulness or cognitive change interventions may inadvertently communicate unhelpful messages such as ‘Just stop feeling bad’ or ‘Just think positively’. To mitigate iatrogenic harm, interventions that identify symptoms or diagnoses should offer related skill-building and help youths formulate helpful and problem-solving thoughts. In fact, several interventions offer skills to improve mental health in adolescents without explicitly raising awareness of psychiatric symptoms or disorders; examples include Amaka Amasanyufu in Uganda^3^ and Shamiri in Kenya.^4^

The article notes that a small percentage of students may deteriorate as a result of discussing negative feelings with peers in group settings. This is certainly possible, as is the opposite: students may benefit from hearing their peers’ positive thoughts or relating to their peers’ struggles. It is likely that both are true, and qualitative research may help clarify the nature and frequency of helpful and harmful comments by peers and facilitators, informing effective structuring and leadership of groups.

Regarding universal interventions, our own school-based research on universal interventions for Kenyan adolescents revealed clinically reliable worsening in 12.42% of participants for depression and 11.78% for anxiety symptoms from pre- to post-intervention (Venturo-Conerly et al^5^ and unpublished data). Interestingly, these rates are comparatively lower than estimates seen in previous research on clinical populations. This suggests that universal interventions may not consistently be more harmful than interventions for populations with elevated symptoms, especially when considering statistical artefacts such as floor effects.^6^ In addition, data collection and scoring and identifying those who meet clinical criteria are major logistical hurdles, particularly in settings with few electronic devices or unreliable internet.

The article cautions that school-based mental health interventions are not inherently better than nothing. However, the risk of iatrogenic harm is not unique to school-based mental health interventions – virtually no intervention universally produces good outcomes and never causes adverse effects. To do the greatest good, we must develop scalable interventions (e.g. accessible and cost-effective school-based universal interventions) that cause far more good than harm. Future research identifying factors associated with harm could inform how harms associated with scalable, accessible interventions may be minimised, helping to address the youth mental health treatment gap.
